# An exploration of how the disruption of mainstream schooling during the COVID-19 crisis provided opportunities that we can learn from so that we may improve our future relationship with the more-than-human world

**DOI:** 10.1007/s43545-022-00588-1

**Published:** 2023-01-13

**Authors:** Dylan Adams, Tonia Gray

**Affiliations:** 1 Cardiff Metropolitan University, Cardiff, Wales; 2 Western Sydney University, Sydney, Australia

## Abstract

The COVID-19 crisis forced educators globally to reassess delivery of their curricula and educational priorities. Already the worsening climate crisis amid human beings’ deleterious relationship with the more-than-human world has caused many educators to demand radical reconsiderations as to the purpose and meaning of education. We ask: how did the disruption of mainstream schooling, during the COVID-19 crisis, provide opportunities that we can learn from so that we may improve our future relationship with the more-than-human world? We examine research that shows children can benefit from outdoor, contemplative pedagogical approaches that differ from their usual indoor classroom experience. Outdoor, contemplative pedagogical approaches involving freedom from “clock-time”, as were experienced by some children during the lockdowns, can allow for ways of knowing and states of being that are often marginalised or excluded from mainstream schools in industrial growth societies. We draw on underpinning theory that argues the status quo in schools conforms to a factory model of schooling that prioritises limited ways of knowing and states of being, thus, lacking the perspectives needed to enable children to cultivate a sustained, healthy relationship with nature. We suggest that outdoor contemplative approaches are required that allow children to dwell pedagogically and experience their relationship with the more-than-human world.

## Introduction

On the 11th of March 2020, the World Health Organization declared that the spread of the new coronavirus, COVID-19, was a pandemic and governments began to implement non-pharmaceutical interventions. These interventions included stay-at-home orders, curfews, cordons sanitaires, societal restrictions and school closures. Colloquially known as “lockdowns”, these measures meant that, suddenly, the normal day-to-day reality in urban environments had stopped as the roads and streets emptied and all but “essential workers” stayed at home. This situation was described as a global crisis. The word crisis comes from the Latinised form of the Greek *krisis* meaning, “turning point in a disease, that change which indicates recovery or death” (Online Etymology Dictionary 2021). Perceiving times of crises as potential turning points offers a more hopeful future, presenting them as opportunities. We may consider also that the word “opportunity” comes from the Latin phrase “*ob portum veniens*” meaning coming toward a port, and the word “port” figuratively is understood as a “haven, place of refuge, asylum” (Online Etymology Dictionary 2022). Does this global pandemic give us the chance to disembark from harmful itineraries in education, seek refuge and embrace alternative journeys? In this paper, we ask: How did the disruption of mainstream schooling during the COVID-19 crisis provide opportunities that we can learn from so that we may improve our future relationship with the natural world? We analyse how the concepts of liminality, kairotic time and contemplative approaches relate to some children’s experiences during the lockdowns. These analyses demonstrate how outdoor contemplative pedagogical approaches could help children to cultivate healthy relationships with the natural world.

Before the COVID-19 crisis was declared, the world was already faced with an even larger existential threat, in the form of the climate crisis (Quay et al. 2020). The UN's IPCC report on climate change concluded it was “code red for humanity” meaning that unless there is a drastic cut in emissions of greenhouse gases, then rising temperatures and sea levels will cause catastrophic devastation for life on our planet (IPCC 2021). Therefore, in the context of the bigger existential challenge presented by the climate crisis, the COVID-19 crisis could be seen as an opportunity to reset and radically change education in order to heal our relationship with the natural world. As Quay et al. (2020) warn, when the crisis of the COVID-19 pandemic passes, “perhaps the most worrying concern will be if the world is not different, if we just end up reverting to the way things were” (p. 94). Žižek (2021) concurs, arguing that we need to “wake-up” to the realisation that compared to the COVID-19 crisis, global warming demands even more radical measures. Before the COVID-19 crisis, there were already an increasing number of voices calling for changes to mainstream schooling as it is argued the ontological and epistemological philosophies that dominate in classrooms only serve to perpetuate a Western worldview that results in estrangement from and devastation to, the natural world (Bonnett 2020; Gray 2018; Gray and Bailey 2022; Jardine 2000; Jickling et al. 2018; Kincheloe 2008). In this paper, we discuss how the COVID-19 crisis may be presenting us with an insight into pathways that are not only alternative to the status quo in Western education but could be giving us a portal of opportunity that allows for a different trajectory, away from the “factory-model” (Friesen and Jardine 2009; Robinson and Arnica 2016) of schooling and towards greater connection with the other-than-human world.

During the lockdowns, the classroom was replaced with “learning at home” (Drane et al. 2020) for those children who would normally be learning in schools. There was some evidence that during the first lockdown in the UK, children enjoyed more time outdoors and felt more connected to the natural world (Lemmey 2020). However, this did not result in an improved situation for all, as evidence showed that the education of children from poorer families suffered (Andrew et al. 2020, Cowie and Myers 2021; Drane et al. 2020). For example, during the first period of lockdowns in England, it was reported that six per cent of children from poorer backgrounds had no access to any outdoor space (Gilhooly 2020). In addition, children from Black, Asian and Minority Ethnic backgrounds were four times as likely to have no access to outdoor space compared to those from white backgrounds (Gilhooly 2020). Research from Australia similarly reported that school closures due to lockdowns were having a detrimental effect on children, particularly the most vulnerable (Bessell 2021; Brown et al. 2020). Nevertheless, there was evidence that many children experienced an enhanced connection to nature during the COVID-19 crisis due to “having more time to spend outdoors” (Friedman et al. 2021, p.4) and that outdoor learning was particularly valued by those working with the most vulnerable and disadvantaged (Institute for Outdoor Learning 2021). Friedman et al. (2021) quote one parent as explaining that: “Our lives have slowed down and so we notice the tiny things, the growth in plants from one day to the next. It has been a very weird headspace, viral armageddon (sic) on our doorsteps but simple quiet beauty of the natural world in our back garden” (Friedman et al. 2021, p. 4).

There was also evidence that children in Switzerland, Canada, and Estonia enjoyed having more agency over the time and tempo for their learning (Stoecklin et al. 2021). Stoecklin et al. (2021) reported that during lockdowns, children adopted “a slower pace that suited them better” (p.6). Evidence showed some parents valued the increased time they were given with their children as feelings of closeness and bonding improved during lockdown (Lau-Clayton et al. 2020), and many teachers gained a better understanding of their community from supporting home learning (Moss et al. 2020). Of course, if parents stop working from home, the time spent with their children will presumably decrease. However, the change in attitudes may be of lasting benefit. In research from England, 75.7% of urban parents said the lockdowns had caused them to change their attitudes to the importance of access to green space for their children (Howlett and Turner 2022). Research also shows that the lockdowns accentuated a desire to connect to the natural world for children’s improved wellbeing and to improve their relationship with nature (Quay et al. 2020; Pouso et al. 2021; Rios et al. 2021). This has potential significance in relation to the commitment to the United Nations commitment to human and planetary wellbeing (United Nations 2021). Increased contact with, and attitudes towards, the natural world are important factors when we consider how we can combat the climate crisis. Mackay and Schmitt (2019) show that a sense of connection with nature is important for fostering a desire to protect the natural world. As Sobel states, we must allow children “to love the earth before we ask them to save it” (2019, p. 47). Therefore, it is vital that children are given more opportunities outdoors to experience meaningful connections with the natural world.

Trying to identify trends in the experiences of children during the lockdowns is inevitably problematic, however, because of the range of different factors due to geographical and socio-economic circumstances (both between nations and within nations) that impacted on the children’s realities (United Nations 2020). In addition, the timings and circumstances of the different lockdowns and school closures changed depending on each individual nation’s ruling government policy at the time. It is also unlikely that any child experienced wholly positive or wholly negative learning experiences during the lockdowns. As Biesta (2022) has noted, the fears that the COVID-19 has caused a “delay in learning” ignore the fact that “learning didn’t stop” (p. 221) because learning cannot be simply switched on and off. However, the purpose of this paper is not to try to identify generalisable trends that affected children during the lockdowns. Instead, we wish to examine how the disruption of schooling provided opportunities that educators and policy makers can learn from so that we may improve our future relationship with the more-than-human (Abram 1997) world. We believe what is significant is that the COVID-19 crisis has meant many have experienced a seemingly liminal, in-between time, a change to their normal working patterns and modes of existence. This is because liminal experiences can be seen as being portals to improved states of being that could lead to improved relationships with the more-than-human world.

## Liminal opportunities

Liminality is a word coined by the anthropologist van Gennep (1909) from the Latin word, līmen meaning threshold, to describe how rites of passage often involve crossing over a threshold into a new phase of existence. This concept was further developed by Turner (1970) who argued that liminal times were a common feature of different human cultures, occurring when the normal structures of society were suspended or collapsed. Turner argued that liminal times allowed for “reclassifications of reality and man’s relationship to society, nature and culture” (2008, pp. 128–129). The COVID-19 crisis could be viewed as a liminal time as it caused a severe disruption to people’s normal everyday existence. Research reported many people found a renewed sense of kinship with the natural world (Friedman et al. 2021; ONS 2021; Vimal 2022; Venutolo-Mantovani 2021). There has also been a chorus of voices from different countries extolling the benefits of outdoor learning, not only to combat the spread of COVID-19, but also as a long-term endeavour (Burke et al. 2021; Hayes 2021; Mulholland and O’Toole 2021; Spiteri 2020; Stoecklin et al. 2021). Evidence showed that many children enjoyed more time learning outdoors during the liminal period of “lockdowns” (Bubb and Jones 2020; Friedman et al. 2021; Lemmey 2020). Furthermore, children did not want to return to prolonged sitting in classrooms when they returned to school (BBC 2020; Bubb and Jones 2020). Conversely, increased “screen-time”, due to more time spent staring into digital devices, had a detrimental impact on children’s health (Khan and Smith 2020; Seguin et al. 2021). This is unsurprising when we consider the damaging impact of staring into a digital screen on children’s sleep patterns (Olteanu-Pascal and Nadejda 2020) and the way blue light disconnects us from the rhythms of our planet (Stevens et al. 2013; Touitou et al. 2016). This disconnect exacerbates a disorientation that is already well-established for many in the Western world (Shepard 1998). Orr states that “place is nebulous to educators” as we are “a deplaced people for whom our immediate places are no longer sources of food, water, livelihood, energy, materials, friends, recreation, or sacred inspiration” (Orr 2013, p.184). This sense of displacement is not due to environmental disaster, though a lack of connection to place may be helping to create ecological devastation. It is argued that modern societies are beholden to the capitalist appetite for excessive production and consumption and, therefore, we willingly allow our sense of place to be lost if it means we can produce and consume on a larger scale (Smith 2014). Our willingness is maintained by values and structures that chain education to market-based principles, convincing us this is the way things must be, so that our schools feed the market-based economy. (Jardine 2012; Smith 2014).

It is important to reiterate that we are not suggesting that children’s experiences of the lockdowns were wholly positive. As discussed above, there is evidence that shows children’s wellbeing suffered due to increased screen time during the lockdowns (Khan and Smith 2020; Seguin et al. 2021). However, it is argued that outdoor, slower, contemplative approaches can immerse children in natural places (Adams and Beauchamp 2020) and potentially provide a counter-pedagogy to the “sensory overload” (O’Donnell 2015, p. 187) that children suffer from due to excessive time spent on digital devices. This slowing down can allow children to “dwell authentically” in natural environments “without interruption or distraction” (Foran and Olson 2012, p. 198). Crinall et al. (2020) argued that the lockdowns caused a revaluation via “a reconceptualising of identity” that entailed “a shift in ontology to re-consider time” (p. 67). Far from being dystopian, they considered the new rhythms of homeschooling as providing opportunities to know “life and existence without the impost and certainty of clock time as our only beat” (Crinall et al. 2020, p. 78). Crinall et al. (2020) reflect on their experience of the combination of lockdown and homeschooling, describing how this allowed them to move with rhythms more in tune with the natural world. They argue this experience of “no time” exists when “the human body is being guided by movements with/as nature” (Crinall et al. 2020, p. 77). Being connected to the rhythms of nature involves being in a “bodyplacetime tempo” (Crinall et al. 2020, p. 68), being willing to be spontaneous, not tied down to future commitments to specific actions at specific times. This “no time” (Crinall et al 2020) is related to Murris and Borcherd’s “childing”, the time of play, an experience of “being in time or always in process” (2019, p. 21). This resonates also with Griffiths’ (2013) “wild time”, a time that experiences “such irrefutable now-ness and this-ness where everything is lit with its innerness” (p. 341). This is neither the time of the past nor the present, but rather is the time of the eternal now, where clock time dissolves in feelings of unity and abundance (Griffiths 2013). This is the time of kairos, the Greek concept of time that is different to the clock time of chronos (Merriam and Webster 2021). Kairos is the opportune moment or “critical time” (Sampson 2020, p. 772). It is “the special moment, the colourful, variegated time of the psyche” (Griffiths 2013, p. 115). It is when something happens “that cannot happen just at ‘any time,’ but only at that time” (Smith 2002, p.47), it is felt as a full appreciation of the moment. While *chronos* is horizontal, linear time, perpetually moving forward, *kairos* is the vertical time of now in its fullness, a deliberate dwelling in the moment and experiencing a sense of eternity (Griffiths 2013). The COVID-19 crisis brought with it a change to the normal day-to-day reality that seems to have been experienced and valued by many during the lockdowns as it provided enhanced wellbeing improved connection to the natural world and better balance to their lives (Friedman et al. 2021; Venutolo-Mantovani 2021). This outcome resonates with Dickson and Gray (2022) who contend that contemporary education requires more nature-rich experiences to ensure a more sustainable future. They argue that outdoor, nature-rich experiences could be a salve to many of the detrimental effects of COVID-19 crisis due to the potential benefits to people’s wellbeing. It is important to stress that we are not suggesting that the COVID-19 crisis has introduced the idea that an increase in outdoor, nature-rich experiences will help improve children’s relationship with nature. Rather we wish to explore how some children’s positive experiences during the COVID-19 crisis allowed for an enhanced relationship with the more-than-human world.

These experiences are significant as they resonate with the non-temporal dimension of kairos that Sampson (2020) describes as “due-measure”. This is not a quantitative measurement but rather is “the qualitative measure of something that is ‘just right’” (Sampson 2020, p. 775). Feelings of enhanced wellbeing, closeness and bonding between parents and children, or teachers and their community, or people and the natural world, are felt qualitatively rather than measured quantitatively. These more balanced existences were arguably at least partly afforded due to a freedom from clock time (Crinall et al. 2020; Friedman et al. 2021; Venutolo-Mantovani 2021). It seems that, for many, experiences of *kairos* replaced living under *chronos.* The COVID-19 crisis has meant that many have experienced a change to their normal daily patterns and modes of existence. The disruption to the normal patterns of schooling for many children has involved working at home. This disruption can be viewed as a seemingly liminal, in-between time that reveals positive potential, as it offers valuable pedagogical perspectives that are alternative to those currently prevalent in mainstream schools of societies in the West. If we are to show how the lockdowns provided a sense of liminality and opportunities for an improved relationship with nature in comparison to the status quo in schools, it is important that we analyse how the normal dominant pedagogical approaches in industrial growth societies have come to inhibit a harmonious relationship with the more-than-human world.

## The dominance of clock time

It is argued that one of the most significant factors inhibiting change and maintaining the grip of the dominant culture in education is the perspective, and use, of time (Jardine 2013; Seidel 2014; Smith 2006). As Adam (2013) explains, the “norms for sequencing and prioritising in education are located in very specific if implicit, theories of time” (p. 66). Mainstream classroom education in the West is dominated by allegiance to “clock-time” (Seidel 2014). Schools are still largely beholden to a factory model of organisation (Jardine 2013) where buzzers and bells sound out the need for new periods of work and “break” times. Pupils often remain in confined class rooms until time has passed on the clock and they are “let out” on the playground or required to move en-masse to a different classroom for a different lesson. This tight clock organisation partly fuels a desire to provide evidence that time spent in the classroom has been worthwhile (Jardine 2008). Jardine states, the “temporality of industrial fragmentation” not only “demands the fragmentation of that which it measures” but also “measures the truth of things by the ability to control, predict and manipulate such fragments” (2008, p. 8). Clock time is, thus, exchanged, mirroring market principles, for measurable impact or evidence of progress. Macy and Brown (2014) use the term “industrial growth societies” to describe modern industry-driven societies and emphasise how clock time is used to help measure and fuel the incessant lust for industrial growth. The technologies and economic forces whipped up by the industrial growth society “radically alter our experience of time, subjecting us to frenetic speeds and severing our felt connection with past and future generations” (Macy and Brown 2014, p. 169). This disconnect is achieved by removing our sense of embodiment as our compulsion to get somewhere else and our focus on screens mean we become numb to the realisation of our earthly connections. Augé (2009) suggests we exist in “non-places” as “in the world of supermodernity people are always, and never, at home” (p. 87). Rifkin argues that our only escape from the anxiety caused by existing in an artificial time world is to return to an “empathetic time world” where we experience “how nature and life unfold over time” (1989, p. 226). He declares that a “revaluation of time is a prerequisite to the revaluation of life” (Rifkin 1989, p. 227).

These perspectives are supported by Virilio (2007, 2008) who analysed how Western society’s slavish devotion to communication technologies can be conceptualised as an obsession with “dromology”, a word he invented that describes the prioritisation, or politics of speed in all considerations of such technologies. Modern societies suffer from “dromospheric pollution” (Virilio 2008, p. 22), as the communication technologies we use involve a pollution of distance, time and place. This unperceived pollution of distances pollutes our relations with others and also our sensory realities as the fullness of these experiences are anesthetised. The physical place we exist in becomes obscured to our senses, polluted due to our fixed attention to digital screens “on a restricted planet that is becoming just one vast floor” (Virilio 2008, p. 23). The “here” of the “here and now” is eliminated as the communications revolution means that a “teletopical metacity is constructed around the window and the teleport, that is to say around the screen and the time” (Virilio 2008, p. 26). The feeling of time itself is impoverished as we experience a “time freeze” giving way to a “world freeze” that is “an imperceptible withholding of the world’s extension and of its regional diversity” (Virilio 2008, p. 32). This theorising seems particularly prophetic and relevant to our examination of mainstream education when we relate it to the virtual communications that increasingly dominate the educational landscape since the onset of the COVID-19 crisis. Evidence suggests that children’s screen time increased to the detriment of their wellbeing (Seguin et al. 2021; Ribner et al. 2021). But these concerns also potentially are applied to post-pandemic education. Children are increasingly required to complete homework tasks, many of which will be completed via digital technology, being on the internet and staring at screens (Korhonen 2021; Mullan 2019). The virtual world is a de-placed world, that is, it removes us from our sense of place as we exist virtually in no place. We surf, disembodied, in cyberspaces that ever weaken our sense of embodiment and “the significance of physical location” (Berman 2004, p. 60). When “on screens” we are not concerned with here, as our only concern becomes getting to some future deficit time severed from place. Even our “now” is corrupted, only partially experienced as our lust for a future moment of task completion takes over, dulling us to the fullness of time (Jardine 2013; Smith 2006). Smith (2006) analyses how this manifests itself in the mantra of “when you complete this (course, grade, assignment, year, etc.) then you can…” (p. 25). Thus, the dominant pedagogy mirrors market logic whereby an exchange in the present is made for a future product. This causes education to exist in a kind of “frozen futurism” as what was expected to be revealed has already been revealed (Smith 2006). Therefore, “education seems like a preparation for something that never happens because, in the deepest sense, it has already happened” (Smith 2006, p. 25). Moreover, the frenzied tempo of industrial growth societies must be maintained in schools lest we should fall behind (Smith 2006). What results is an incessant, unrelentingly fast-paced classroom terrorising teachers and students alike (Gray and Pigott 2018). As Seidel (2014) explains, educational institutions seem to be “rushing ahead of themselves” (p. 120), perpetually desirous of a certain future and to arrive as quickly as possible, marching at “a furious and inhumane pace” and consuming us all with promises of “progress and forward motion” (Seidel 2014, p. 120). Jardine (2013) echoes the feelings of educators the world over in industrial growth societies who have become accustomed to trying to keep up with the pace of modern schooling yet experience the feeling that time is always running out. Jardine (2013) analyses how this vicious and cyclic predicament has its roots in the “efficiency movement” (Callahan 1964) that grew from Taylor’s (1911) “Principles of Scientific Management”. Taylor declared “in the past the man has been first; in the future the system must be first” (Taylor 1911, p. 1) and set about devising and justifying a system for optimising efficient production from manual work in factories. This became so popular that it influenced the organisation and management of industrialised nations, so “the uniformity, standardization, and bureaucracy of the factory model soon became predominant characteristics of the school district” (Jardine 2013, p. 10). It is argued that this “factory-model” of schooling has become only more entrenched in industrial growth societies due to an over-obsession with measurable targets and standardised exams (Robinson and Aronica 2016). Under this regime of “efficiency”, students become “raw material transported along the educational assembly line” (Jardine 2013, p. 10). Even the idea of “engagement” becomes “caught in regimes of market-exchange”, where students engage to “receive marks that can then be exchanged for future employment that then can be exchanged for one’s chosen enjoyments” (Jardine 2013, p. 13). Moreover, “this sense of relentless, perpetually running-out machine time” has become so commonplace that it is experienced “not as something that has emerged under causes and conditions that can be otherwise, but as if it is simply ‘the way things are’” (Jardine 2013, p. 7) Jardine reminds us that this is not inevitable, rather “it is how the world has turned out” (2013, p. 7) and that alternatively “there is thoughtfulness, rigorousness, authenticity and good work to be had” in alternatives to “this running- out panic” (2013, p. 9).

## A new story is needed

Where and how are we to find these alternatives? Berry (2015) argues humankind needs a new story and claims our fate is dependent on the stories we believe and follow. Our old stories of how we fit into the world are antiquated, and we need a new narrative of hope to ameliorate the damage caused by anthropocentric dominance. He asserts this anthropocentrism “is largely consequent on our failure to think of ourselves as a species” (Berry 2015, p. 21). The COVID-19 crisis and the climate crisis, however, are perhaps forcing people to acknowledge we are members of the same species. Of course, we are not suggesting that everyone is living out the same story. All human beings on the planet are facing the challenge of living with the COVID-19 virus, but not everyone is facing the same level of individual challenges. Nevertheless, COVID-19 did, at least momentarily, stop most people in their tracks, and as Žižec (2021) suggests, “it is only through this mortal threat that we can envision a unified humanity” (p. 105). There is a long lineage of voices (Arendt 2006; Dewey 1923; Freire 1985; Kincheloe 2011) who have argued that to set right the world we must undertake a “rewriting” or re-storying of our place in the world. Hope is needed so we may dream these stories into existence. Change cannot take place unless we are free to dream and imagine new stories, and hope is required “as there is no dream without hope” (Freire 1992, p. 81).

After emerging from the crisis of the Second World War, Arendt (2006) claimed that education should prepare our children “in advance for the task of renewing a common world” (p. 193). She argued that education can create stories of hope as “education is the point at which we decide whether we love the world enough to assume responsibility for it” (Arendt 2006, p. 193). Freire (2001) similarly recognised that the liminal call of crises can be answered by the hopeful stories of education. We can see this in the hopeful experiences of Crinall et al., who experienced “a reconceptualising of identity” that entailed “a shift in ontology to re-consider time” (2020, p. 67) during lockdown while homeschooling their children. They state that the lockdown “opened up a nurturing learning space” where their children thrived and “endless time” seemed available (Crinall et al. 2020, p. 71). Their willingness to be spontaneous and be guided by the rhythms of nature and the intuitive energies of their children was rewarded with an enhanced feeling of connection as a family and in community with the natural world. As has been discussed above, this feeling is echoed in the research of Friedman et al. (2021) who found that most children and their parents had experienced a greater sense of community with each other and the natural world during “lockdowns”, leading to increased feelings of wellbeing. These experiences of community with the natural world are not prioritised in mainstream education in the West (Quay et al. 2020). Instead, educational experiences generally reflect the dominant culture in modern industrialised societies whereby “we have deluded ourselves into assuming human progress and civilization depends on dominating and transcending nature” (Quay et al. 2020, p. 99). Consequently, the disconnection between nature and children is becoming increasingly apparent and mainstream education is only exacerbating the problem (Gray 2018; Jickling et al. 2018). Abram (2010) warns that “our sensory encounter with the ambiguous depth of our world” is deteriorating, due to “our steady involvement with flat representations of that world” (p. 12). As we retreat behind the screens of various digital technologies, our sensory experience of the moment becomes impoverished and perhaps our abilities to feel multisensory encounters in the future diminish (Abram 2010). Despite this, there is some evidence that suggests outdoor learning in natural environments is gaining global momentum (Mann et al. 2022). The potency of nature-rich teaching and learning is being underpinned by evidence-based research which suggests a myriad developmental and wellbeing benefits for children and adolescents (Kahn and Kellert 2002; Mann et al. 2022). Furthermore, giving children these experiences could help achieve the United Nations’ sustainable development goals (SDGs) as evidence suggests people who feel more connected to nature will do more to protect it (Mackay and Schmitt 2019). Simply replicating the factory model of schooling outdoors may not provide the potency of experience that is required to make a positive impact on children’s sense of relation with the natural world (Adams and Beauchamp 2020; Beauchamp et al. 2022) Evidence shows that it is the quality of experiences that children need, rather than just an extended length of time outdoors (Richardson et al. 2022). However, facilitating the types of experiences required seemingly runs counter to the priorities of mainstream education (Jardine 2012; Jickling 2017; Quay et al. 2020).

That our understanding of, and relationship with the more-than-human world (Abram 1997) needs changing, and this involves a transformation of education, is not a new idea. Many have previously called for a radical change to the “metaphysics of education” (Bonnett 2004) so that children are able to learn *in* and *from* the other-than-human world (Evernden 1999; Jardine 1998; Smith 2006; Jickling et al. 2018). It is argued that we need to re-consider not only the purpose of education, but also the kinds of knowledge and values around which it should be orientated, and “thus of the fundamental attitude towards the world that it should convey” (Bonnett 2004, p. 11). Jardine (2012) argues we suffer from the delusion that “if bureaucratically diligent enough” we can “assure a future with no risk or uncertainty, no need for further thinking or negotiation or venture” that we can simply replace bad solutions with good solutions like “some sort of error that can be and needs to be fixed” (p. 4) without questioning the ontological and epistemological assumptions that dominate the world view of industrial growth societies. As Seidel explains, “without wisdom, ‘knowing’ has no purpose other than creating more knowledge” (2014, p. 141) and, thus, merely plays into the paradigm of working for working’s sake, producing knowledge for the knowledge economy, continually “constructing learners as workers” (Seidel, 2014, p. 141).

## Different ways of knowing

The type of knowledge or ways of knowing that are prioritised in schools in industrial growth societies involve objective, logical, and rational thinking (Jardine 2012). Objective thought is raised up as being superior to all other ways of knowing as it is believed to entail a purity free from any outside influences (Bordo 1987). Within this paradigm, this type of knowing, or “reason”, becomes the judge and jury of all things and is able “to demand that the Earth must henceforth live up to the clarity of distinctness requisite of mathematization” (Jardine 2000, p. 89). Bordo warns if “the whole of nature were only what can be explained in terms of mathematical relationships, then we would look at the world with that fearful sense of alienation, with that utter loss of reality with which a future schizophrenic child looks at his mother” (1987, p. 97). We examine and come to know the world “through abstraction, through labelling, separating it from us, through taking apart to understand” (Hart 2019, p. 339). This type of knowledge involves a narrowing of focus in order to seek “precision, detail, and objectivity” (Hart 2019, p. 339). This disembodied rational logic not only denies our interrelatedness with the more-than-human world, but it also ignores other ways of knowing. It ignores the way we can know things through the body, through our senses, through our imagination, through our intuitions, through our feelings, through relational group experiences and through contemplative practices (Miller et al. 2019). In the arts, for example, contemplative knowings (the imagination, feelings, and intuitions) are often far more important than rational ways of knowing (Miller et al. 2019). Hart (2004) describes contemplative knowing as involving the “capacity for deepened awareness, concentration, and insight” (p. 29). Yet generally in schools in the West, these ways of knowing are marginalised in comparison to logical, rational ways of knowing (Miller et al. 2019). In indigenous cultures, contemplative ways of knowing are far more respected than in Western educational practices (Cajete 2019; Miller et al. 2019). Therefore, we can view the dominance of so-called “rational” ways of knowing as a type of epistemic colonialism that has resulted from a Western ethnocentric view of the world (Kincheloe 2008) prioritised in the factory model of schooling (Robinson and Aronica 2016).

A recurring theme in Traditional Indigenous Knowledge (TIK) is the notion of *dadirri* or deep listening. This form of nature attunement and introspective practice is at the epicentre of Indigenous custom. Aboriginal elder Miriam Rose Ungunmerr Baumann, and Senior Australian of the Year in 2021, explains the concept of *dadirri* as follows:

To know me is to breathe with me.

To breathe with me is to listen deeply.

To listen deeply is to connect.

It is the sound of deep calling to deep.

Dadirri is the deep inner spring inside us.

We call on it and it calls on us.

(cited in Thomas et al. 2018, pp 163–164).

This theme chimes with Hart’s (2004) concept of “deep listening”, a type of listening that differs from passive or active listening. Deep listening involves listening to the outer and inner world being attentive to our inner feelings, our bodily sensations and our imagination (Hart 2004). These ways of knowing exist in the time of kairos as they involve dwelling in the eternal now and recognising the differences between “the swings of morning, the embers of evening and all the hours in between” (Griffith 2013, p. 115).

## Contemplative approaches allow for connection

Smith (2014) provides hope for our pedagogical futures by calling on us to learn from ancient wisdom traditions and value contemplative practices as portals to more relational ways of knowing and holistic states of being. Contemplative activities help cultivate this attentive awareness as they play out in the time of kairos, and students who take part in them can feel more connected to each other and the other-than-human world (Adams and Beauchamp 2021; Barrable et al. 2021; Gray and Mitten 2018; Hart 2004; Miller et al. 2019). It is argued that contemplative practices can act as portals to more relational ways of knowing and holistic states of being (Beauchamp et al. 2022; Miller 2018; Smith 2014). Therefore, contemplative activities in schools could help children to recognise their interdependence with, and existence as part of, the natural world (Barrable et al. 2021; Hart 2004; Miller et al. 2019). Contemplative, mindful practices involve an epistemology that understands and celebrates the “essential unity of the world” and our “lived interdependence” (Smith 2014, p. 36). Seidel and Jardine (2014) highlight that contemplative teaching is reactive, moving “to the rhythms of life, to what is happening now” (p. 182). This approach provides a radical critique of Western education (Ergas 2015) as contemplative teaching is prepared to wonder, “is willing to doubt” and “to be uncertain” (Seidel and Jardine 2014, p. 182). Therefore, “we learn to attend to - and expect - anything, rather than something that we have already decided upon” (Pulkki 2015, p. 24). This stance is not motivated by some indulgent laissez-faire attitude, but rather “slowing down and being here brings us face to face with the implications of ignoring our connectedness” (Seidel and Jardine 2014, p. 181). Contemplative approaches are not beholden to pre-determined outcomes and “planning everything ahead of time”; thus, they make space “for children’s lives and children’s bodies” (Seidel and Jardine 2014, p. 182).

Contemplative approaches can take place indoors of course, but it is argued that when this “slow pedagogy” takes place outdoors it can result in an “ecocentric intercorporeality” whereby there is a felt bodily connection with the more-than-human world (Payne and Wattchow 2009, p. 14).

These approaches involve “dwelling pedagogically” (Foran and Olson 2012, p. 198) in sensory perception and afford children a “patient receptivity” (Abram 1997 , p. 30) to their embodied experience of place and themselves. This body consciousness is important as it allows children to sense being part of the more-than-human world, thus, moving from an “ego-consciousness” (Pulkki et al. 2017, p. 40) to an eco-consciousness. Pulkki et al. (2017) argue that alienation from the physical environment leads to environmental devastation. Cultivating body awareness and enhanced sensory capabilities could enable a “biophilia revolution” where children experience feeling part of the natural environment (Pulkki et al. 2017, p. 214). Freeing ourselves from the “ontological delusion” that we are individuated and separated from each other and the more-than-human world allows us to experience “the dependent co-arising of things and the dependent co-arising and shaping of our-selves in light of this insight” (Jardine 2012, p. 17). Miller (2018) argues that we need to foster the holistic development of children by cultivating this “love” for the more-than-human world. Therefore, children will experience the more-than-human world as a “communion of subjects rather than a collection of objects” (Miller 2018, p. 63) and feel part of this communion. Contemplation, thus, becomes an act of love “where we live with things” and “there is no them but only us” (Miller 2018, p. 91).

Research has shown engaging children in contemplative activities in nature reserves can produce enhanced understandings and appreciations of their relationship with the natural world (Adams and Beauchamp 2020, 2021; Beauchamp and Adams 2022). Evidence suggests that children can have augmented ways of knowing that involve feeling an expanded sense of self and heightened sense of connection with the more-than-human world (Adams and Beauchamp 2020, 2021). Teachers and children reported that freedom from clock time was a key part of these contemplative approaches and the children’s experiences (Adams and Beauchamp 2020, 2021; Beauchamp and Adams 2022). One of the children describes feeling as though “time didn’t matter” (Adams and Beauchamp 2021, p. 11), and all the children reported that this freedom from clock time was joyfully experienced. The children also reported feeling a sense of freedom from the normalities of school and a sense of immersion in the nature reserve. For example, one of the children stated that they cannot usually hear the trees and the birds “because the school’s in the way” (Adams and Beauchamp 2020, p. 134). A sensory feeling of “otherness” had seemingly allowed the children to feel *in* and *part of* the natural world (Adams and Beauchamp 2020, 2021). Yet the noisiness and fast pace of normal school life usually prevent this feeling of immersion and communion (Adams and Beauchamp 2020, 2021; Beauchamp and Adams 2022).

## Conclusion

In the introduction to this paper, we asked: How did the disruption of mainstream schooling provide opportunities that we can learn from so that we may improve our future relationship with the more-than-human world? Sobel (2019) argues that before we ask children to save the world, we must afford them experiences that enable them to love the world. Children need experiences that allow for moments, not minutes, in nature (Richardson et al. 2022). Evidence shows that immersive, contemplative experiences in nature places can afford children with alternative ways of knowing and augmented states of being that involve feeling as one with the natural world (Adams and Beauchamp 2020, 2021; Beauchamp and Adams 2022). We suggest that the COVID-19 crisis has given us the opportunity to halt our harmful trajectories in education and renew our thinking and practices. This liminal time has allowed many children to slow down, engage in more contemplative practices and reconnect with each other and the natural world (Crinall et al. 2020; Friedman et al. 2021; Lemmey 2020; Vimal 2022). Of course, for others, this period has merely increased their hardships (Whitehead et al. 2021), and there is little evidence that there will be a willingness to alter curricula after the COVID-19 crisis. If this is to be a liminal time that leads to an improved sense of community with the more-than-human world, then radical changes are needed to the pedagogical approaches of mainstream schools in industrial growth societies. We argue that only by experiencing a different sense of reality from that which is predominantly enacted in schools in industrialised societies will children be able to cultivate a sustainable empathetic relationship with the more-than-human world. Schools need to foster love for the natural world through outdoor, contemplative, kairotic experiences that give children an enhanced relationship with nature. Evidence suggests that this will ensure future ecologically harmful practices will diminish (Mackay and Schmitt 2019), thus, helping to combat the climate crisis and helping to achieve the United Nations’ SDGs. The COVID-19 crisis has the potential, therefore, to be our turning point away from curricula that prioritise narrow ways of knowing and states of being, so that we may improve our relationship with the more-than-human world.

## Data Availability

Data sharing not applicable to this article as no datasets were generated or analysed during the current study.
